# Bronchial Progenitor Cells in Obstructive and Neoplastic Lung Disease: A Pilot Study

**DOI:** 10.3390/jcm13020609

**Published:** 2024-01-21

**Authors:** Beatrice Ragnoli, Federica Fusco, Patrizia Pignatti, Tiziana Cena, Guido Valente, Mario Malerba

**Affiliations:** 1 Respiratory Unit, S. Andrea Hospital, 13100 Vercelli, Italy; beatrice.ragnoli@aslvc.piemonte.it; 2 Laboratory of Pathology, Az. Ospedaliera Maggiore della Carità, 28100 Novara, Italy; 10036274@studenti.uniupo.it; 3 Allergy and Immunology Unit, Istituti Clinici Scientifici Maugeri IRCCS Pavia, 27100 Pavia, Italy; patrizia.pignatti@icsmaugeri.it; 4 Epidemiological Observatory Service, ASL VC, 13100 Vercelli, Italy; tiziana.cena@aslvc.piemonte.it; 5 Laboratory of Pathology, Department of Traslational Medicine, School of Medicine, University of Eastern Piedmont, 28100 Novara, Italy; guido.valente@med.uniupo.it

**Keywords:** chronic obstructive pulmonary disease, basal cells, airway remodeling, smoking, epithelial–mesenchymal transition, airway fibrosis, lung cancer, bronchial progenitor cells, neoplastic lung disease, p63

## Abstract

The alteration of progenitor/stem cells present in the airway epithelium has been observed in patients with COPD. Smoking exposure induces remodeling patterns in bronchial progenitor cells (BPCs), encompassing squamous metaplasia, hyperplasia of basal and of mucus-secreting cells, and the depletion of ciliated and non-mucous secretory cells. Our aim was to assess the expression of p63 and vimentin as potential markers of airway remodeling and the regulation of stem cell populations in obstructive and neoplastic lung disease patients. A retrospective single-center observational study was conducted, including patients undergoing bronchoscopy with bronchial biopsies for suspected lung cancer. p63 and vimentin expression were evaluated via immunohistochemical analysis. There were 25 patients, of which 21 with COPD were included, and 17 were diagnosed with lung cancer. We observed that FEV1% was negatively correlated with p63+ basal cell number (r = −0.614, *p* = 0.019) and positively correlated with vimentin expression (r = 0.670; *p* = 0.008). p63 was significantly higher in biopsies from the trachea and main bronchi compared to more distal areas (*p* = 0.040), whereas vimentin was prevalent in the more distal areas (*p* = 0.042). Our preliminary data suggest the initial evidence of structural changes in BPCs among patients with COPD and lung cancer. Further research efforts are warranted to investigate additional morphologic and functional respiratory parameters in these patients.

## 1. Introduction

The natural history of chronic obstructive pulmonary disease (COPD), the primary smoking-induced lung disorder, spans several decades and is marked by a significant and continuous decline in forced expiratory volume in the first second (FEV1), a hallmark of airway obstruction [1]. 

Airway remodeling, a major histologic characteristic of airway obstruction in COPD patients [2,3], has been observed even in the early stages of the disease and in individuals who smoke and exhibit COPD-like symptoms despite experiencing only a minor decrease in FEV1 [4,5,6]. Thus, there may be an early junction in the developmental course of COPD where the homeostatic regenerative mechanisms responsible for preserving airway structure integrity become ineffective. Alternatively, these mechanisms may start triggering tissue remodeling, which serves as the morphological basis for airflow limitation. Indeed, these homeostatic mechanisms have been shown to play a significant role in maintaining the integrity of the airway epithelium, which is continuously exposed to environmental stressors, including cigarette smoke. 

The main function of the progenitor/stem cells in the epithelium of the respiratory tract is to provide airway homeostasis and to repair defects in the airway wall. 

Bronchial progenitor/stem cells (BPCs) in the proximal airways are recognized as resident stem cells capable of self-renewing and differentiating to virtually every pseudostratified epithelium cell type under steady-state and after acute injury. BPCs, residing within the basal epithelial layer alongside basal cells (BCs), are responsible for generating all cellular components of the airway epithelium. These include BCs, which are produced via self-renewal, ciliated cells, mucus-secreting cells, and non-mucous secretory cells. Upon exposure to smoking, BPCs initiate remodeling patterns, encompassing squamous metaplasia, hyperplasia of BCs and mucus-secreting cells, and the depletion of ciliated and non-mucous secretory cells [7]. Of note, the reduced functionality of BPCs has been observed in smokers with COPD [8]. Whether this phenomenon occurs at an earlier stage in the natural progression of COPD remains an open question.

One potential mechanism contributing to airway fibrosis involves the transition of airway epithelial cells into a mesenchymal phenotype with myofibroblast characteristics. These cells subsequently migrate into the lamina propria, a process referred to as epithelial–mesenchymal transition (EMT) [9,10].

Studies conducted on biopsies obtained from large airways of smokers and individuals with COPD have shown a dynamic process of EMT in these airways, further corroborated by the identification of reticular basement membrane fragmentation in both current and former smokers with COPD. Moreover, a significant increase in the staining of epithelial BCs for epidermal growth factor receptor (EGFR), fibroblast-specific protein 1 (S100A4), and matrix metallopeptidase 9 (MMP-9) across all smoking or ex-smoking groups [11,12,13,14] was reported. 

Epithelial cells typically undergo a transition, losing their epithelial characteristics, including polarity and junctional proteins, to acquire mesenchymal features, such as vimentin filaments [15]. In a recent study, vimentin-expressing epithelial cells were analyzed as key markers of EMT-related de-differentiation. These cells were found to be increased in both large and small airways of COPD patients. In addition, the expression of vimentin in the epithelium correlated with airway obstruction, as judged by the post-bronchodilator FEV1 and FEV1/vital capacity (VC) ratio [16].

P63 is mainly expressed in cells of the basal layer where high regenerative processes occur. Its expression can be altered in tumor cells. Epithelial differentiation and their derangement into tumor cells can be balanced by p63 expression [17]. 

In this scenario, the transcription factor p63, which triggers the termination of cell division or apoptosis in response to DNA damage [18], is known to play a crucial role in sustaining stem cell populations in squamous and other stratified epithelia [19]. This peculiar function is further supported by its detectable presence in bronchial reserve cells [20].

This pilot study was designed to identify structural changes in BPCs among obstructive and neoplastic lung disease patients. Our goal was to assess the expression of p63 and vimentin as potential markers for airway remodeling and regulation of stem cell populations.

## 2. Materials and Methods

### 2.1. Population

We conducted a retrospective single-center observational study, including all patients undergoing bronchoscopy with bronchial biopsies for suspected lung cancer between the years 2017–2018 at the Lung Unit of Sant ‘Andrea Hospital in Vercelli, Italy. This study was approved by the Institutional Review Board CE 67/20 in accordance with the principles of the Declaration of Helsinki. An informed consent was obtained.

Peripheral lung cancers were excluded from the case series, resulting in the selection of 33 patients. We collected the histological diagnosis of lung tumor, the localization of lung lesions on CT scan, the presence or absence of emphysema, respiratory function test results, and COPD diagnosis from each participant’s medical history.

We obtained lung function test data for 13/33 patients at the beginning of the study. For patients with the finding, on CT scans of centrilobular emphysema, we retrospectively collected historical lung function tests confirming that all patients with emphysema also had a chronic obstructive disease. 

The exclusion criteria comprised the presence of any of the following characteristics: (i) history of any cardiovascular disease, excluding arterial hypertension (AH); (ii) diabetes mellitus; (iii) COPD exacerbation or respiratory infections in the previous 6 months; and (iv) a diagnosis of asthma.

### 2.2. Respiratory Function Tests

Lung spirometry was performed following standard procedures. Forced vital capacity (FVC) and FEV1 were measured with the subject seated, and the spirometry maneuvers were repeated at least three times to obtain the best results. Percent-of-predicted values were calculated using the data standards provided by the ATS/ERS guidelines [21]. 

### 2.3. Biopsies and Methods of Investigation 

Bronchial biopsies were collected as part of the bronchoscopic examination. Specifically, we obtained samples from suspected endobronchial and adjacent mucosal neoplasms, which were then rapidly fixed in 10% neutral buffered formalin for at least 18 h and embedded in paraffin. From these specimens, 4 μm sections were precisely cut using a microtome and then mounted on Superfrost slides. After that, the sections were stained with hematoxylin–eosin (H&E). 

Following a histologic review, eight cases were excluded due to an inadequate representation of the bronchial epithelium. This resulted in the inclusion of 25 out of the initial 33 cases in the study. 

Immunohistochemical analysis (IHC) for p63 and vimentin was performed on sections from the selected cases using a Ventana–Roche automated immunostainer. These markers, namely p63 (4A4 clone) and vimentin (V9 clone), were established in previous studies for detecting BPCs and EMT, respectively [15,19,20].

The expression of p63 and vimentin was evaluated at high magnification using a Leica DM 2500 light microscope connected to an LG monitor. The assessment consisted of counting the number of positive cells among the total number of epithelial cells, with at least 200 cells examined for each case, while ensuring that vimentin-positive leukocytes were excluded. The values were expressed as percentages on total cells. All slides were anonymously coded to blind the analyst.

### 2.4. Statistical Analysis 

Data were compiled by considering mean and standard deviation (SD) for normally distributed continuous variables and using absolute and relative frequencies for categorical variables. Differences in mean expressions for each variable were assessed using either Student’s *t*-test for normally distributed variables or Mann–Whitney’s U-test. Data normality was assessed via the Shapiro–Wilk test. Pearson’s correlation test was carried out to explore the relationship between the positivity rates of p63 and vimentin and the percentage values of FEV1. 

All *p*-values were calculated as 2-tailed (two-way test), and a *p*-value < 0.05 was considered statistically significant. All statistical analyses were performed using GraphPad Prism 7 (GraphPad Software, San Diego, CA, USA).

## 3. Results

### 3.1. Patients

Table 1 displays the demographic characteristics of the patients included in the study. Overall, 5/33 patients were diagnosed with COPD with lung function tests at the beginning of the study. Then, on the CT scans, the presence of centrilobular emphysema was detected in 16 out of the selected 25 patients (64%). For these patients, we retrospectively collected historical lung function tests. This adjustment increased the number of COPD diagnoses to 21 out of 25 patients (84%).

The majority of patients were former smokers (20/25). As expected, COPD patients showed impaired FEV1% predicted mean values compared to non-COPD patients (78.3 ± 19 vs. 91.7 ± 10.2). There were no notable differences in blood pressure (BP) values among treated patients for AH with and without COPD. Overall, 17/25 patients (68%) were diagnosed to have lung cancer. The majority of lung cancer patients (13/17) had COPD with a mean FEV1% predictive mean value of 80.4 ± 10. In our sample, among lung cancer hystotypes, the most represented were squamous cell carcinoma (6/17) and adenocarcinoma (6/17). 

### 3.2. p63 Expression

**Figure 1:**
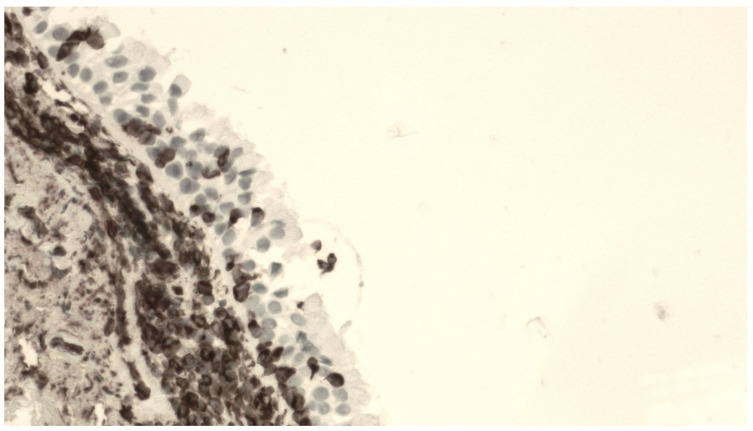
Fragment of bronchial mucosa stained by an anti-p63 antibody. A number of positive cells (corresponding to progenitor basal cells) are distributed along the base of the epithelium at 400× magnification.

The mean number of p63+ BPCs was 36.6 ± 7.3 cells. Figure 1 shows a representative staining of bronchial biopsies for p63 expression.

We found an inverse correlation between FEV1% of predicted values and the number of p63+ BCs (r = −0.614, *p* = 0.019) (Figure S1). The correlation between p63+ bronchial cells and FEV1 expressed in liters was also statistically significant (r = −0.55 and *p* = 0.041) (Figure S2). 

The amount of p63+ cells was comparable between patients with COPD and those without (Table 2). Patients with emphysema did not differ from patients without emphysema in terms of p63+ cell count (36.84 ± 6.85 vs. 36.34 ± 7.82, respectively; *p* = 0.86). 

No differences in p63+ cell counts were found among patients with different histologic tumor types (i.e., large cell carcinomas, squamous cell carcinomas, adenocarcinomas, or small cell carcinomas). However, the localization of the p63+ cells was significantly higher in biopsies from the trachea and main bronchi compared to more distal areas (*p* = 0.040) (Table 2).

### 3.3. Vimentin Expression

**Figure 2:**
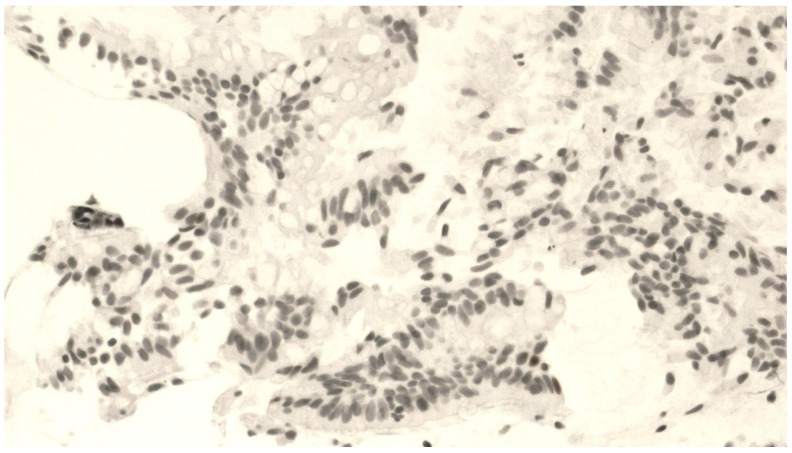
Fragment of bronchial mucosa immunohistochemically stained by an anti-vimentin antibody. The presence of an epithelial–mesenchymal transition is shown by a number of positive cells distributed inside the epithelium at 400× magnification.

**Figure 3:**
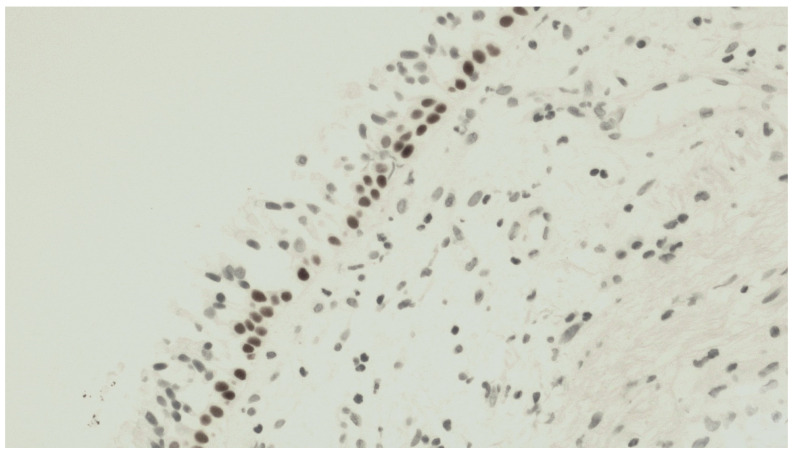
Control of immunohistochemical staining omitting the primary antibody: absence of staining at 400× magnification.

The mean number of vimentin+ cells was 21.8 ± 9.5 cells. Figure 2 shows a representative staining of bronchial biopsies for vimentin expression. In Figure 3, it is reported that a control of immunohistochemical staining omitted the primary antibody and was documented by the absence of staining.

In contrast to p63, we found a positive correlation between FEV1% predicted and vimentin expression (r = 0.670; *p* = 0.008; Figure S3), whereas no significant correlation was observed between vimentin+ cells and FEV1 when the latter was expressed in liters (r = 0.13 and *p* = 0.64; Figure S4). Similarly, there were no discernible differences in vimentin expression between COPD and non-COPD patients (Table 3). No difference was found between emphysematous and non-emphysematous patients (21.65 ± 10.96 vs. 20.71 ± 6.73, respectively, *p* = 0.80). 

Finally, in marked contrast with p63, vimentin expression was more prevalent in the more distal areas (lobar and segmental bronchi) compared to the more proximal ones (=0.042; Table 3).

## 4. Discussion

Despite the limited number of cases included in this study, we report some noteworthy preliminary findings. We found an inverse correlation between the number of p63+ BCs and FEV1% values. This suggests that the expression of this marker for BPCs increases as lung function declines, according to FEV1 measurements. Conversely, p63+ cell counts seem to decrease when lung function is preserved. This finding aligns with the established role of BCs in the literature [22], which involves regenerating all cellular components of the bronchial epithelium in the presence of damage [23]. As lung function deteriorates, these cells gain a more prominent role in re-establishing the epithelial barrier, thereby protecting the airway from pathogens [24]. In contrast, in the absence of disease, when FEV1% is within normal ranges, the lower number of p63+ cells we found may represent a state of epithelium stability without the need for active cell turnover. This correlation remained consistent when we considered FEV1 in absolute values rather than as a percentage. We did not record significant differences between patients with and without emphysema. 

Another significant finding in this study was the correlation between vimentin expression and FEV1% values. As reported in the literature, EMT is a reversible process whereby a polarized epithelial cell is transformed into a cell with a mesenchymal phenotype [25]. This transformation is associated with increased migratory capacity, invasiveness, resistance to apoptosis, and production of extracellular matrix components [26]. Due to these characteristics, EMT is associated with malignancy in various cancer types, and the activation of EMT signaling in cancer cells is widely recognized for its contribution to metastasis, recurrence, or the development of therapeutic resistance [27]. The results we obtained may therefore be attributed to the fact that patients within the study population underwent bronchoscopy to investigate the nature of their lesions, which, in almost all cases, were found to be cancerous. Thus, the observed increase in vimentin, along with a normal/high FEV1% value, could be driven by the central role of EMT in the development and progression of carcinomas, as also suggested by data in the literature [25,26,27]. 

Furthermore, COPD patients frequently present airway fibrosis and remodeling characterized via EMT [28]. Vimentin is primarily involved in EMT processes via the reduction in cell adhesion and enhancement of the ability of cells to migrate [29]. In the bronchial epithelium of COPD patients, vimentin expression is related to the thickening of the basement membrane [16]. Exposure to cigarette smoke seems to be one of the major determinants of EMT in bronchial epithelial cells via activation of the TGF-β1/Smad pathway [30,31].

Another important observation pertains to the differing expressions of both vimentin and p63 at the various biopsy sampling sites. Specifically, we found that p63+ cells were more abundant in the trachea and main bronchi than in the lobar and segmental ones, whereas the reverse pattern was found for vimentin. This difference may be attributed to purely anatomical factors. Indeed, the distribution of cells in the epithelium is tightly controlled: BCs are prevalent throughout the conducting epithelium, but their number declines with decreasing airway caliber [32]. Over time, these cells become progressively rarer and are gradually replaced by Clara cells, which also have progenitor functions [33]. This would then account for the reduced occurrence of p63 positivity in samples from lobar and segmental bronchi compared to those from the trachea and main bronchi. 

To our knowledge, at the time of writing, in the literature, there are only a few animal models on this topic. One study showed that p63-expressing basal cells are less represented in the distal region of the airways both in humans and in mice. These data confirm our results regarding the higher expression of p63 in biopsies from the trachea and main bronchi compared to more distal areas [34,35]. The “in vitro” p63 expression was evaluated in bronchial epithelial cell cultures and bronchial epithelial lung explants from both smokers without COPD and patients with COPD. In both conditions, p63 expression decreased after three culture cycles. The decline in the amount of progenitor/stem cells, as confirmed by decreased p63 expression, suggests that p63 might be involved in the mechanism of tissue repair of the airway epithelium in COPD patients [36]. Vimentin expression was higher in a mouse model of COPD induced by cigarette smoke exposure than in mouse controls. The authors found the same results with primary bronchial epithelial cells co-cultured with cigarette smoke extract in the presence of IL-17A, suggesting bronchial epithelial–mesenchymal transition [37]. 

Moreover, with aging, pulmonary functional and structural deterioration occurs, mainly in pathological conditions, which is associated with impaired stem cell activity and increased senescence in mice. However, the impact of these processes underlying lung physiopathology in relation to aging has not been explored in humans. Recent innovative research analyzed stem cell, senescence, and proliferative markers in lung samples from young and aged individuals, with and without pulmonary pathology. The authors identified a non-reduction in p63+ basal cells in small airways with aging. On the contrary, they revealed an increase in p63+ cells, specifically in aged individuals diagnosed with pulmonary pathologies. These results provide new evidence of the activity of p63+ stem cells on human lung regeneration and point out that regeneration machinery in the human lung is activated under stress due to aging but fails to repair in pathological cases, as stem cells would likely become senescent [38].

Our study presents some limitations: first of all, the retrospective nature of the study; moreover, being a pilot study, the sample size is very small and does not allow for the comparison of different subgroups of patients; finally, it is not possible to evaluate the effect of smoking being almost all patients’ former smokers. 

## 5. Conclusions

In conclusion, our study establishes significant associations between BPCs and EMT, as indicated by their respective immunohistochemical markers and respiratory function parameters. While these results are based on a limited sample size, they provide initial evidence of structural changes in BPCs among patients with COPD and lung cancer. 

In light of these findings, we believe that further research efforts are warranted to investigate additional morphologic and functional respiratory parameters in this patient category. Future investigations should be aimed at assessing the differential expression of p63 and vimentin in patients with normal lung function (as measured by FEV1 and FEV1/FVC) and imaging patterns of emphysema, potentially indicating a specific COPD phenotype. In addition, the evaluation of their differential expression in patients with or without COPD and with or without emphysema may offer other valuable insights. Finally, a more comprehensive assessment of EMT could be achieved by employing other immunohistochemical markers.

## Figures and Tables

**Table 1:** Characteristics of the enrolled patients.

	Enrolled Subjects N = 25
Age (mean ± SD), years	73.6 ± 8.1
Sex M/F	15/10
Non-smokers/Former smokers	5/20
FEV1% predicted (mean ± SD)	78.3 ± 19
COPD with E	16/25 (64%)
COPD without E	5/25 (20%)
Lung cancer	17/25 (68%)
Squamous cell carcinomas	6
Adenocarcinomas	6
Small cell carcinomas	3
NSCLC	2

COPD = chronic obstructive pulmonary disease, E = emphysema, FEV1 = forced expiratory volume in the first second, SD = standard deviation, NSCLC = non-small cell carcinomas.

**Table 2:** p63-positive bronchial cell expression in different groups of patients.

P63+ Bronchial Cells		
	Mean	*p*
COPD	36.79 ± 6.64	0.68
Non-COPD	35.24 ± 8.96	
Biopsies from the trachea or main bronchi	36.69 ± 6.72	0.04
Biopsies from lobar or segmental bronchi	34.00 ± 6.72	

**Table 3:** Vimentin expression in different groups of patients.

Vimentin+ Bronchial Cells		
	Mean	*p*
COPD	21.54 ± 10.56	0.96
Non-COPD	21.76 ± 5.34	
Biopsies from the trachea or main bronchi	17.32 ± 6.95	0.04
Biopsies from lobar or segmental bronchi	24.62 ± 10.02	

## Data Availability

The data presented in this study are available by the authors. For any further request contact the corresponding author.

## Supplementary Materials

The following supporting information can be downloaded at https://www.mdpi.com/article/10.3390/jcm13020609/s1. Figure S1: representation of FEV1% predicted and p63+ bronchial cells (PBC) in COPD patients; Figure S2: representation between FEV1 L and p63+ bronchial cells (PBC) in COPD patients; Figure S3: representation between %FEV1 predicted and vimentin in in COPD patients; Figure S4: representation between FEV1 L and vimentin in in COPD patients.

## References

[1] Vestbo J., Lange P. (2016). Natural history of COPD: Focusing on change in FEV1. Respirology.

[2] Hogg J.C., Chu F. (2004). The nature of small-airway obstruction in chronic obstructive pulmonary disease. N. Engl. J. Med..

[3] McDonough J.E., Yuan R., Suzuki M., Seyednejad N., Elliott W.M., Sanchez P.G., Wright A.C., Gefter W.B., Litzky L., Coxson H.O. (2011). Small-airway obstruction and emphysema in chronic obstructive pulmonary disease. N. Engl. J. Med..

[4] Regan E.A., Lynch D.A. (2015). Clinical and Radiologic Disease in Smokers with Normal Spirometry. JAMA Intern. Med..

[5] Woodruff P.G., Barr R.G. (2016). Clinical Significance of Symptoms in Smokers with Preserved Pulmonary Function. N. Engl. J. Med..

[6] Kirby M., Tanabe N. (2018). COLD Collaborative Research Group and the Canadian Respiratory Research Network. Total Airway Count on Computed Tomography and the Risk of COPD Progression: Findings from a Population-based Study. Am. J Respir. Crit. Care Med..

[7] Shaykhiev R., Crystal R.G. (2014). Early events in the pathogenesis of chronic obstructive pulmonary disease: Smoking-induced reprogramming of airway epithelial basal progenitor cells. Ann. Am. Thorac. Soc..

[8] Staudt M.R., Buro-Auriemma L.J. (2014). Airway basal stem/progenitor cells have diminished capacity to regenerate airway epithelium in chronic obstructive pulmonary disease. Am. J. Respir. Crit. Care Med..

[9] Jeffery P.K. (2004). Remodeling and Inflammation of Bronchi in Asthma and Chronic Obstructive Pulmonary Disease. Proc. Am. Thorac. Soc..

[10] Willis B.C., duBois R.M. (2006). Epithelial Origin of Myofibroblasts during Fibrosis in the Lung. Proc. Am. Thorac. Soc..

[11] Kalluri R., Weinberg R.A. (2009). The basics of epithelial–mesenchymal transition. J. Clin. Investig..

[12] Zeisberg M., Neilson E.G. (2009). Biomarkers for epithelial–mesenchymal transitions. J. Clin. Investig..

[13] Kalluri R. (2009). EMT: When epithelial cells decide to become mesenchymal-like cells. J. Clin. Investig..

[14] Ward C., Forrest I.A. (2005). Phenotype of airway epithelial cells suggests epithelial to mesenchymal cell transition in clinically stable lung transplant recipients. Thorax.

[15] Puchelle E., Zahm J.M. (2006). Airway epithelial repair, regeneration, and remodeling after injury in chronic obstructive pulmonary disease. Proc. Am. Thorac. Soc..

[16] Gohy S.T., Hupin C. (2015). Imprinting of the COPD airway epithelium for dedifferentiation and mesenchymal transition. Eur. Respir. J..

[17] Yoh K., Prywes R. (2015). Pathway Regulation of p63, a Director of Epithelial Cell Fate. Front. Endocrinol..

[18] Levine A. (1997). p53, the cellular gatekeeper for growth and division. Cell.

[19] Yang A., McKeon F. (2000). p63 and p73: p53 mimics, menaces and more. Nat. Rev. Mol. Cell Biol..

[20] Senoo M., Pinto F. (2007). p63 is essential for the proliferative potential of stem cells in stratified epithelia. Cell.

[21] Miller M.R., Boggs P.B. (2005). Standardisation of spirometry. Eur. Respir. J.

[22] Crystal R.G., Randell S.H. (2008). Airway Epithelial Cells, Current Concepts and Challenges. Proc. Am. Thorac. Soc..

[23] Smirnova N.F., Schamberger A.C. (2016). Detection and quantification of epithelial progenitor cell populations in human healthy and IPF lungs. Respir. Res..

[24] Crosby L.M., Waters C.M. (2010). Epithelial repair mechanisms in the lung. Am. J. Physiol. Lung Cell. Mol. Physiol..

[25] Jonsdottir H.R., Arason A.J. (2015). Basal cells of the human airways acquire mesenchymal traits in idiopathic pulmonary fibrosis and in culture. Lab. Investig..

[26] Otsuki Y., Saya H. (2018). Prospects for new lung cancer treatments that target EMT signaling. Dev. Dyn..

[27] Jolly M.K., Ward C. (2018). Epithelial-mesenchymal transition, a spectrum of states: Role in lung development, homeostasis, and disease. Dev. Dyn..

[28] Su X., Wu W., Zhu Z., Lin X., Zeng Y. (2022). The effect of epithelial-mesenchymal transitions in COPD induced by cigarette smoke: An update. Respir. Res..

[29] Usman S., Waseem N.H., Nguyen T.K.N., Mohsin S., Jamal A., Teh M.-T., Waseem A. (2021). Vimentin Is at the Heart of Epithelial Mesenchymal Transition (EMT) Mediated Metastasis. Cancers.

[30] Shen H., Sun Y., Zhang S., Jiang J., Dong X., Jia Y., Shen J., Guan Y., Zhang L., Li F. (2014). Cigarette smoke-induced alveolar epithelial-mesenchymal transition is mediated by Rac1 activation. Biochim. Biophys Acta.

[31] Pan K., Lu J., Song Y. (2021). Artesunate ameliorates cigarette smoke-induced airway remodelling via PPAR-γ/TGF-β1/Smad2/3 signalling pathway. Respir Res..

[32] Knight D.A., Holgate S.T. (2003). The airway epithelium: Structural and functional properties in health and disease. Respirology.

[33] Reynolds S.D., Malkinson A.M. (2010). Clara cell: Progenitor for the bronchiolar epithelium. Int. J. Biochem. Cell Biol..

[34] Rock J.R., Onaitis M.W., Rawlins E.L., Lu Y., Clark C.P., Xue Y., Randell S.H., Hogan B.L.M. (2009). Basal cells as stem cells of the mouse trachea and human airway epithelium. Proc. Natl. Acad. Sci. USA.

[35] Rock J.R., Hogan B.L.M. (2010). Branching takes nerve. Science.

[36] Bostancieri N., Bakir K., Kul S., Eralp A., Kayalar O., Konyalilar N., Rajabi H., Yuncu M., Yildrim A.Ö., Bayram H. (2023). The effect of multiple outgrowths from bronchial tissue explants on progenitor/stem cell number in primary bronchial epithelial cell cultures from smokers and patients with COPD. Front. Med..

[37] Chu S., Ma L., Wu Y., Zhao X., Xiao B., Pan Q. (2021). C-EBPβ mediates in cigarette/IL-17A-induced bronchial epithelial-mesenchymal transition in COPD mice. BMC Pulm. Med..

[38] Moreno-Valladares M., Moncho-Amor V., Silva T.M., Garcés J.P., Álvarez-Satta M., Matheu A. (2022). KRT5+/p63+ Stem Cells Undergo Senescence in the Human Lung with Pathological Aging. Aging Dis..

