# Supplementary material for: Bronchial Progenitor Cells in Obstructive and Neoplastic Lung Disease: A Pilot Study
Source: J Clin Med. 2024 Jan 21;13(2):609. doi: 10.3390/jcm13020609 (PMC10816161; doi:10.3390/jcm13020609)
Supplement: Supplementary file 1 [file jcm-13-00609-s001.zip › jcm-2697598-supplementary.pdf]

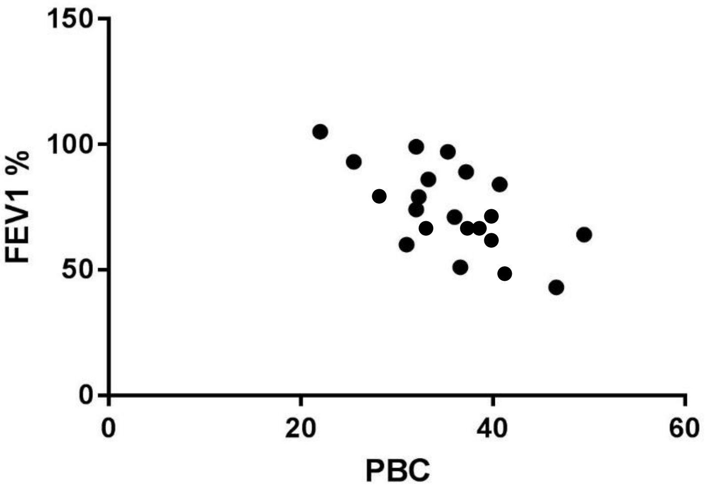

Figure S1. Biplot representation of %FEV1 predicted and p63 + bronchial cells (PBC) in COPD patients.

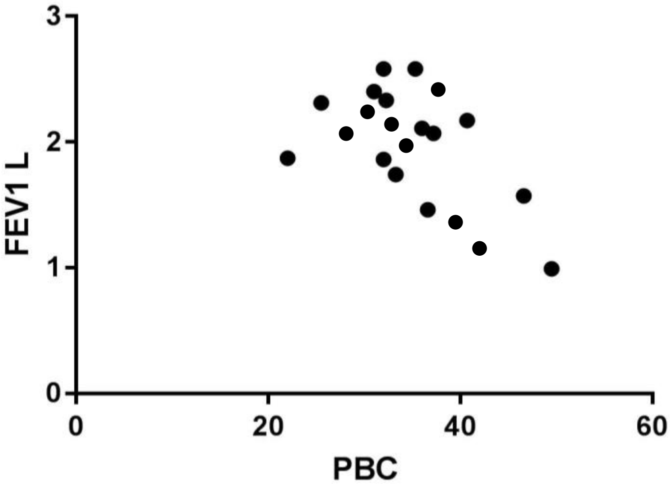

Figure S2. Biplot representation between FEV1 L and p63 + bronchial cells (PBC) in COPD patients.

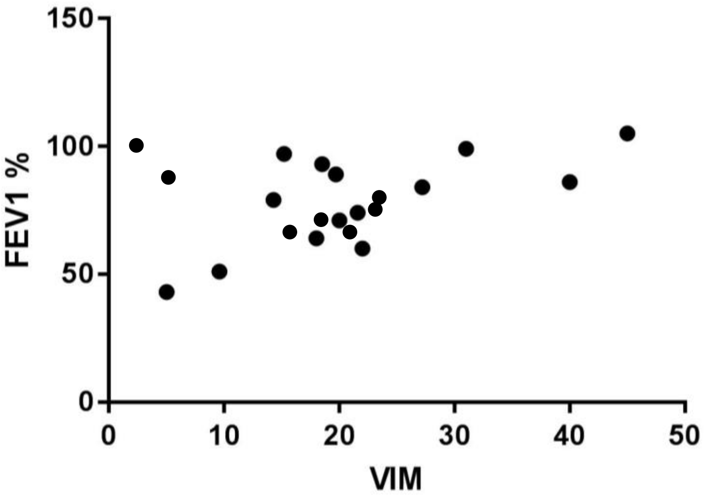

Figure S3. Biplot representation between %FEV1 predicted and vimentin in COPD patients.

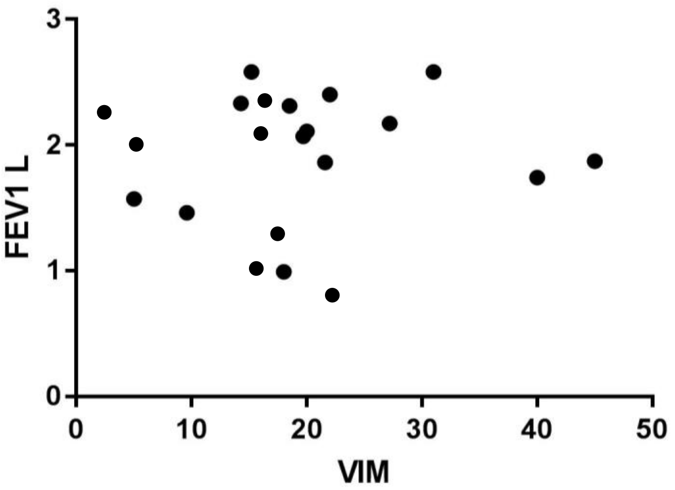

Figure S4. Biplot representation between FEV1 L and vimentin in COPD patients.
